# Cellular fluid shear stress on implant surfaces—establishment of a novel experimental set up

**DOI:** 10.1186/s40729-017-0085-3

**Published:** 2017-05-31

**Authors:** P. W. Kämmerer, D. G. E. Thiem, A. Alshihri, G. H. Wittstock, R. Bader, B. Al-Nawas, M. O. Klein

**Affiliations:** 1 Department of Oral and Maxillofacial Surgery, Facial Plastic Surgery, University Medical Centre Rostock, Schillingallee 35, 18057 Rostock, Germany; 2 Department of Prosthetic and Biomaterial Sciences, King Saud University, Riyadh, Saudi Arabia; 3 Harvard School of Dental Medicine, Boston, MA USA; 4 Department of Oral and Maxillofacial Surgery, Plastic Surgery, University Medical Centre Mainz, Mainz, Germany; 5 Department of Orthopedics, University Medical Centre Rostock, Rostock, Germany

**Keywords:** Bioengineering, Biomechanics, Dental implant materials, Implant healing, Cell biology, Osteoblast, Stress analysis

## Abstract

**Background:**

Mechanostimuli of different cells can affect a wide array of cellular and inter-cellular biological processes responsible for dental implant healing. The purpose of this in vitro study was to establish a new test model to create a reproducible flow-induced fluid shear stress (FSS) of osteoblast cells on implant surfaces.

**Methods:**

As FSS effects on osteoblasts are detectable at 10 dyn/cm^2^, a custom-made flow chamber was created. Computer-aided verification of circulation processes was performed. In order to verify FSS effects, cells were analysed via light and fluorescence microscopy.

**Results:**

Utilising computer-aided simulations, the underside of the upper plate was considered to have optimal conditions for cell culturing. At this site, a flow-induced orientation of osteoblast cell clusters and an altered cell morphology with cellular elongation and alteration of actin fibres in the fluid flow direction was detected.

**Conclusions:**

FSS simulation using this novel flow chamber might mimic the peri-implant situation in the phase of loaded implant healing. With this FSS flow chamber, osteoblast cells’ sensitivity to FSS was verified in the form of morphological changes and cell re-clustering towards the direction of the flow. Different shear forces can be created simultaneously in a single experiment.

## Background

Cells can be influenced by different mechanostimuli, which lead to an activation of cellular and inter-cellular responses. These reactions may be caused by either a direct stimulation of the cell body (mechanoreception) or indirect cellular stimulation (response) [1–3]. Extracellular fluid movement induces fluid shear stress (FSS) that can result in different cellular processes including proliferation, migration and gene expression [4].

There are two different ways of cell stimulation by FSS, where both lead to extracellular signalling. First, fluid-induced cell stimulation occurs when the cell surface is in direct contact with the moving extracellular fluid as seen in the vascular endothelium. Second, it has been hypothesised that indirect stimulation occurs via fluid flow through the lacunar network as seen in bones such as close to loaded dental endosseous implants [1–3]. This extracellular cell stimulation leads to an altered cell morphology as well as altered intracellular signal cascades such as changed gene and protein expression pattern [4–7]. A reorganisation of actin fibres in accordance with the flow direction could be observed as well [8].

To prove the theory of a FSS-triggered effect on different cell lines, several in vitro investigations using different flow chambers were conducted [5, 9–12]. In osteoblasts, biochemical responses on FSS in form of an increased intracellular calcium production [13–15] and an increased release of prostaglandins were reported [15–19]. FSS stimulation of osteoblasts also improved the cell adhesion by enhancing the affinity of intracellular integrins to extracellular matrix ligands as well as to biomaterial surfaces [20, 21]. Shear forces’ triggered effects on osteoblasts could be detected at a value of 10 dyn/cm^2^, which almost reflects the in vivo situation [4, 22, 23]. Todays’ frequently used flow chambers mainly simulate the in vivo formed shear forces. However, it is difficult to ensure the required reproducibility and linear flow conditions. The most distinctive feature of currently used flow chambers is a liquid flow along rigidly fixed cell-bearing surfaces. Some of the above mentioned flow devices are either operating with a constant flow velocity or using pulsating flow profiles, which should be applied in case of analysing blood flow characteristics [9, 24]. Computerised investigations of flow chambers by Anderson et al. [4] have shown that deviating shear forces occur in the same flow chamber after repeating the same experiment twice. Consequently, different results of stimulation and cells response are obtained. Another downside of reported flow chambers is the inability to simultaneously set different shear forces in a single experiment.

Therefore, the aim of the present study was to establish a new cell chamber model for FSS simulation and stimulation. In addition to its ease of use, the reported model in this study should meet the requirements of a simple design, generating reproducible flow characteristics next to laminar flows and clearly defined flow gradients on implant surfaces.

## Methods

### Experimental setup

**Fig. 1:**
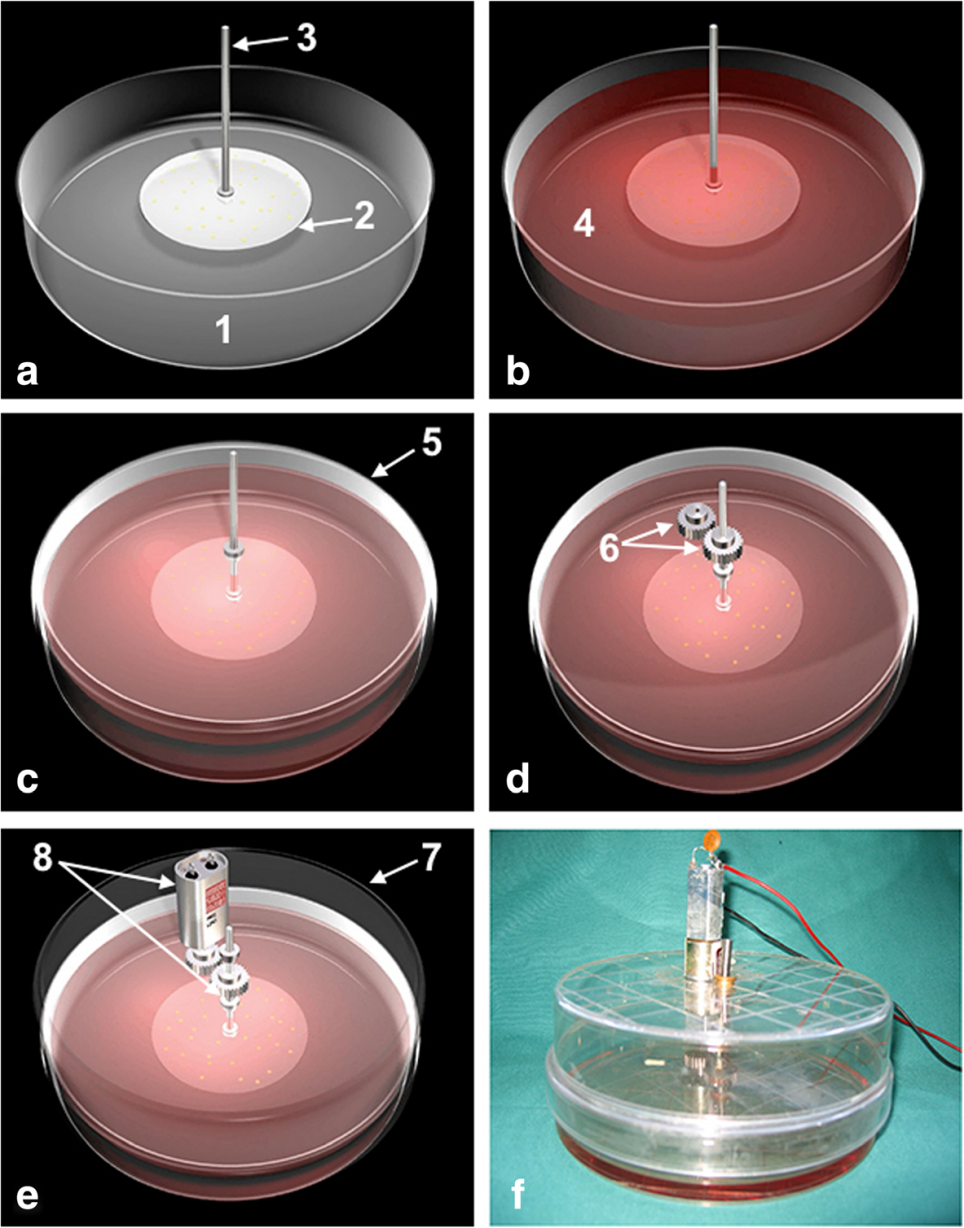
Three-dimensional illustration (**a**–**e**) and photography (**f**) of the experimental setup with the components marked numerical. **a** *1* Lower petri dish (s’ bottom serving as the lower plate); *2* Rotating glass panel [60 mm diameter (cell bearing)]; *3* Titanium axis. **b** *4* Liquid medium (*red*). **c** *5* Reversed upper petri dish. **d** *6* Gearwheel with set screw. **e** *7* Closing; *8* Electronic motor device and adjusting ring with additional set screw

A three-dimensional illustration and photography of the plate/plate flow chamber model is shown in Fig. 1. A detailed list of used parts can be found in Appendix 1.

The circulation within the flow chamber was generated by an externally attached electric motor, which rotates up to 500 rounds per minute (rpm). A commercial grade 4 pure medical titanium gear shaft (length = 40 mm, diameter = 4 mm) was connected to the motor by a set screw-fixed gearwheel. For the attachment of the custom-made biocompatible glass panel (diameter = 60 mm, thickness = 2 mm; 4-mm central circular opening), the gear shafts’ lower end was disc shaped (diameter = 10 mm). The glass panel was fixed by an adjusting ring with an additional set screw from above. The cover of the lower petri dish contained guiding grooves to stabilise an inverted larger petri dish placed on, which in turn served as a holding device to the electric motor (Fig. 1).

### Model assembly

Under sterile conditions, the cell-bearing glass plate was attached to the lower end of the gear shaft with the cells facing the bottom plate. A space of 2 mm between the two plates was determined via computational simulations. After filling the lower petri dish to 70% of its capacity with culture medium, a closing plate with a centred recess for the gear shaft (4 mm) was placed on top to seal the lower compartment. Lastly, a larger petri dish, having a central recess (4 mm), with an externally attached electric motor, was reversely installed above to form the upper compartment. As shown in Fig. 1, the transmission between motor and gear shaft was realised by using two same-sized gearwheels, one fixed on each of the two components within the non-sterile upper compartment.

### Analytical formula for evaluating the flow characteristics

Frequently used flow chambers are characterised by an internal fluid flow along a stationary cell-bearing surface, whereas the osteoblast test cells of this newly developed model are circulating within a resting culture medium.

For constant and fully developed laminar flow between the two parallel plates, the magnitude of the wall shear stress (*τ*) in between was calculated by formula 1:

$$ \tau =\frac{\eta\;r\;\omega}{H} $$

in which *η* is the *dynamic fluid viscosity* (dyn/cm^2^), *r* is the *radius of the plate* (cm), *ω* stands for *angular velocity* and *H* for *height* (*vertical distance in between the two plates*).

To get information whether the flow is laminar or turbulent, Reynolds numbers (Re) were calculated for all flow regimes using formula 2 [25]:

$$ \mathrm{R}\mathrm{e}=\frac{\rho\;v\; A}{\eta} $$

in which *ρ* is the liquids’ density, *η* is its dynamic viscosity, v stands for average angular velocity and *A* is the characteristic area within which liquids flow (vertical distance between the two plates). Re values of >1500 are commonly considered illustrative of turbulent flow as well as values <1500 create laminar flows.

### Computational simulation of the flow characteristics

Numerous computerised simulations were performed to verify flow characteristics occurring within the plate/plate flow chamber assisted by the Department of Hydraulic Machines, Faculty of Mechanical Engineering, Technical University of Munich, Germany. For simulation of flow profiles to assess a potential cellular impact by fluid shear stress inside the chamber, graphical illustrations were created by using Ansys CFX^®^ software (Ansys Germany GmbH, Otterfing, Germany).

### Test procedure

The experimental process involved three steps. First, a count of *n* = 50.000 commercially available osteoblasts (PromoCell, Heidelberg, Germany) per millilitre of culture medium were cultured on the bottom of the cell-bearing surface (glass panel). Therefore, cells were seeded in a culture medium (cf. Appendix 2 for a detailed composition) at 37 °C. Prior to the test procedure, the cells were manually removed from the culture bottles’ bottom by gentle movements while adding 5 ml of Trypsin followed by 10 min of incubation. Finally, Trypsin residues were removed with, first, centrifugation (1600 rpm/5 min) of the cell fluid (culture medium with additives and loose osteoblast cells) and, second, by subsequently adding 10 ml of culture medium. After 24 h of incubation, cells showed adherent to the glass panel. A conventional petri dish was filled to 70% of its capacity with cell fluid (culture medium and additives (Appendix 2). The petri dishs' bottom formed the lower plate and the round glass panel the upper plate placed within the culture medium. Directly after, the circulation process for FSS induction was initiated. In brief, after 24 h of incubation at 37 °C and 5% CO_2_ concentration, cells adhered to under side of the glass panel and the glass panel was incorporated into the device as described above. The circulation process (speed level = 200 rpm) started for 24 h under sterile incubation conditions (37 °C and 5% of CO_2_). Via repeated computational simulations, a rotational speed level of 200 rpm was found as adequate to provide 10 dyn/cm^2^ of shear force at the plates’ peripheral region. Lastly, light microscopic examination was conducted (Leica DC480^®^, Leica Microsystems, Wetzlar, Germany) to verify the cell orientation (after 24 h with and without rotation), followed by phallacidin fluorescence staining according to the manufacturer’s protocol (Appendix 3) (BODYPY® FL Phallacidin, ThermoFisher Scientific, MA, USA) and fluorescence microscopy with Leica/Leitz DM RBE^®^ (Leica Microsystems, Wetzlar, Germany). The cell body and its longitudinal actin fibre orientation was put in relation to the total-force-vector (Fig. 5) (resulted from the flow velocity-vector and the centrifugal force-vector) which was calculated by formula 3. For differentiation into an oriented and non-oriented cell formation, an angle of 90° was set as threshold value.

## Results

Our analysis was focused on two main aspects:

1. Simulation of the fluid flow characteristics as well as quantification of the arising shear forces at the plate/plate flow chamber with reliable reproducibility
2. Assessment of the impact of fluid shear stress on osteoblast cells in terms of altered cell morphology and intracellular structural changes

### Evaluation of the fluid flow characteristics by computerised simulations and quantification of the resulting forces

The computational fluid dynamic analysis and the quantification of the occurring shear forces within the plate/plate flow chamber were central part of this investigation. The cellular-fluid flow setup was based on the fact that osteoblast cells show responses at 10 dyn/cm^2^ at a speed level of 200 rpm.

**Fig. 2:**
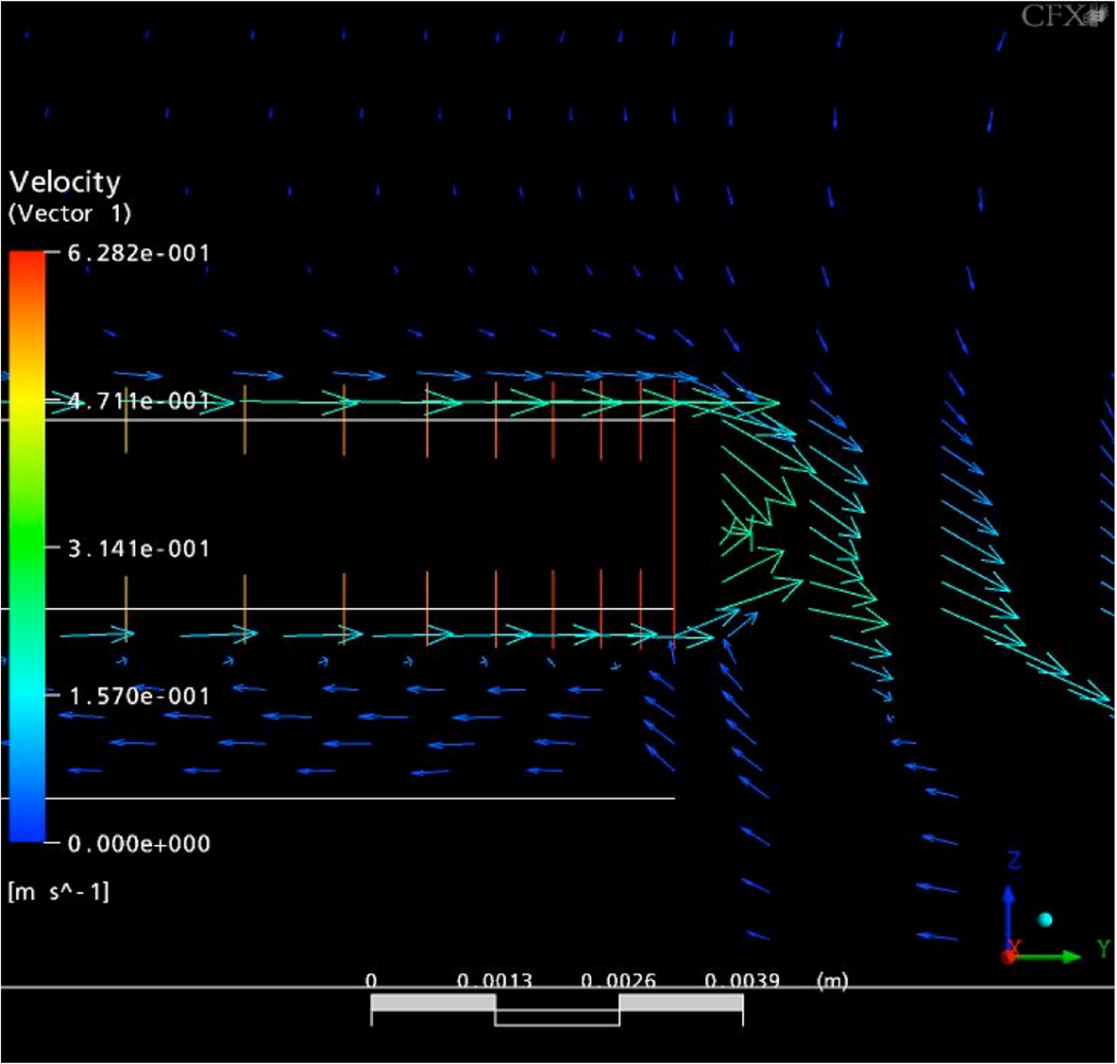
Side view of a computerized simulation, showing the flow chambers’ lower compartment and the flow profile in between the two plates; shearing gap and bottom plate are shown on the *left* side; rotation speed = 200 rpm; colour code bar (*left edge*) showing shear force values [Pa] [1 Pa = 10 dyn/cm^2^]; flow direction presented by *arrows*

On the topside of the upper plate (rotating glass panel), the computerised simulations demonstrated a gradient increased flow with shear forces from the centre (1 dyn/cm^2^) to the periphery (10 dyn/cm^2^). Minor effects of shear forces (0–2 dyn/cm^2^) were recorded on the surface of the bottom plate. It could be demonstrated that a bigger radius of rotation correlates with higher shear forces as expressed in formula 1. Further simulations aimed to verify the pattern of fluid flow (laminar versus turbulent flow). For this purpose, the conditions on the upper and lower surface of the upper plate as well as in the area in between the two plates were of particular interest. The simulations revealed a strong turbulent flow on the entire top surface of the upper plate, especially in the centre and the area around the circumferential edges (Fig. 2). The flow from the peripheral region turned backward and amplified the turbulent flow on the top surface. The development of two opposite flow directions was observed within the area in between the plates. The flow along the lower plates’ surface was directed from the periphery to the centre whereas the fluid movement along the underside of the upper plate was inversely orientated (Fig. 2). Regarding the upper compartment, peripheral turbulent flow along the outer edges was similar to the fluid movements within the area in between the plates. At the top, the turbulent flow directed from the centre to the periphery whereas the turbulences at the bottom were orientated in reverse to that. Moreover, the effect of the shear forces on the osteoblast cells was also influenced by the centrifugal force. This force can be calculated using formula 3:

$$ F = \rho \cdot h\cdot {\varpi}^2\cdot r $$

in which *ρ* = density, *h* = height, *ω* = angular velocity and *r* = radius.

**Fig. 3:**
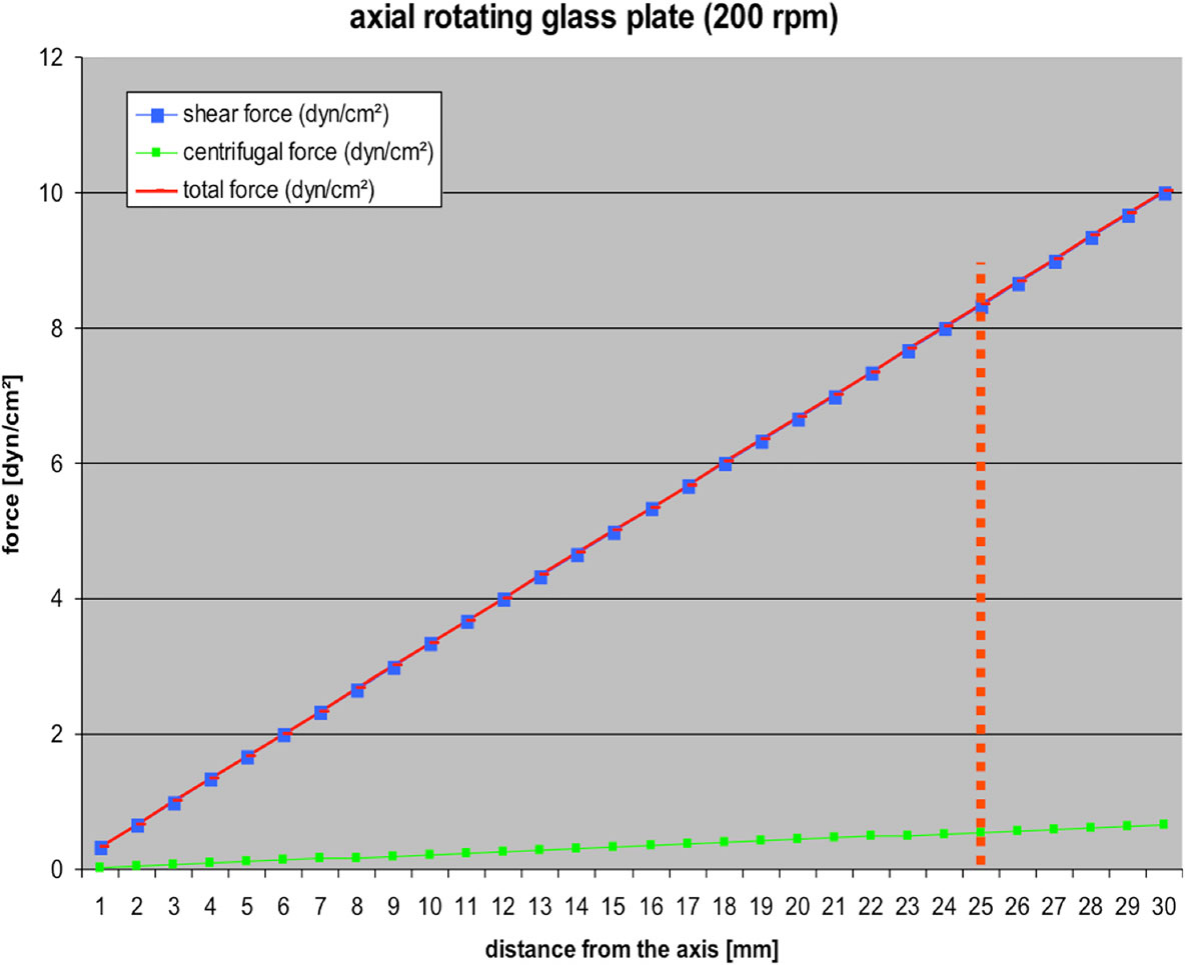
Diagram for visualisation of the calculation of shear stress rates taking into account the centrifugal force and the glass plates’ dimensions. For example, at a distance of 25 mm from the centre of the upper plate, the shear forces’ value is 8.33 dyn/cm^2^, together with an additional centrifugal force that has a value of 0.55 dyn/cm^2^

Figure 3 shows the respective physical force and its dependence on a bigger radius and higher rotational speed. The results of this study indicate that the centrifugal force represents only a little proportion of effective forces. Hence, the centrifugal forces’ impacts on the tested cells are considered to be insignificant.

### Morphological changes of osteoblast cell clusters and individual cells

**Fig. 4:**
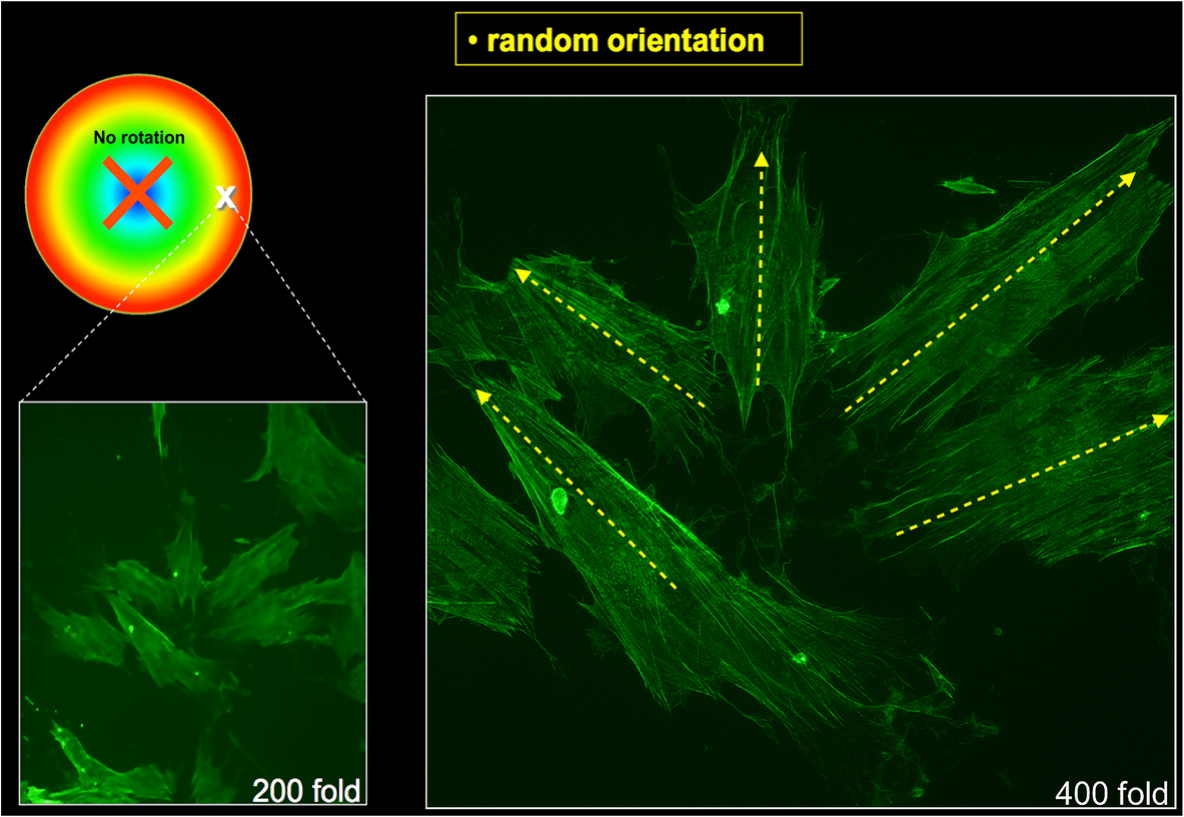
Randomly orientated osteoblasts without influence of rotation (phallacidin fluorescence staining). On the *left side* with 200× and on the *right side* with 400× magnification. The *white* X on the *coloured circle* marks the location upon the plate where the osteoblasts were located. The *red* X marks the centre of the plate

**Fig. 5:**
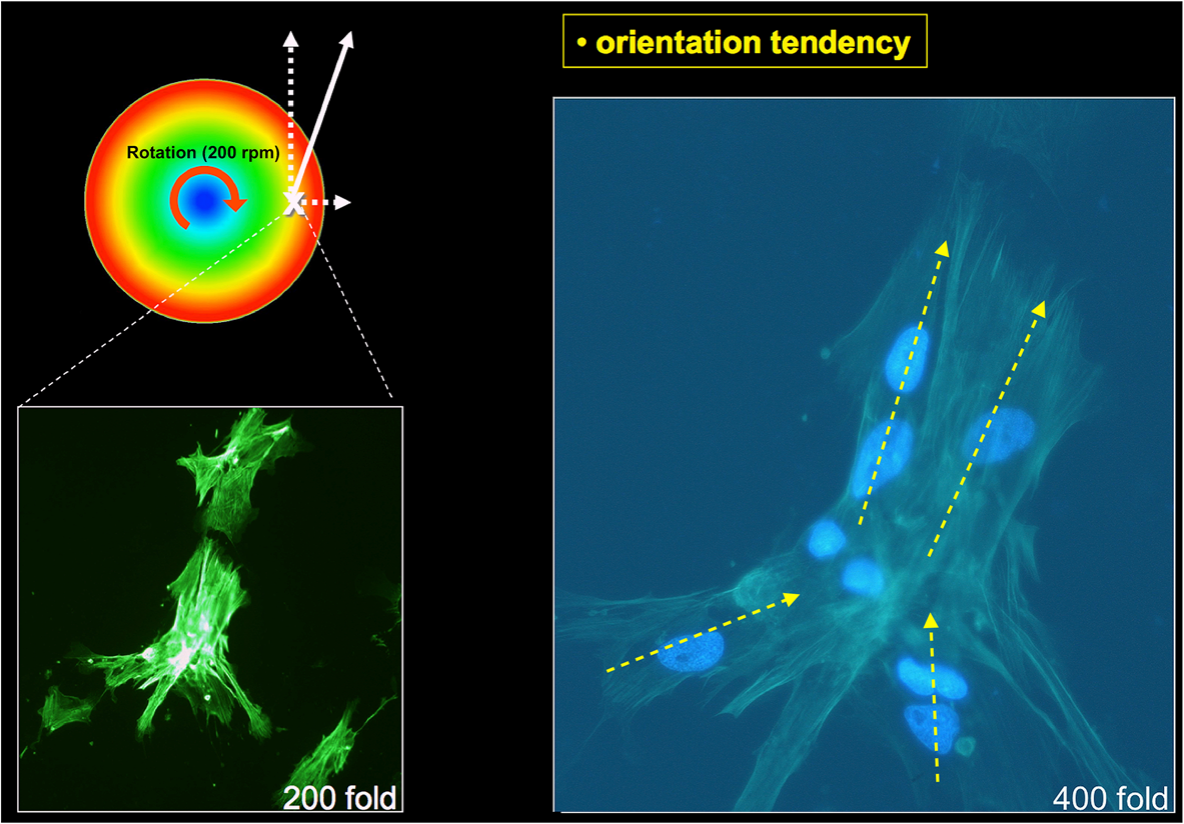
Osteoblasts with an orientation tendency after 24 h of rotation (phallacidin fluorescence staining). On the *left side* with 200× and on the *right side* with 400× magnification. The *yellow arrows* show the orientation of the cells. The *red arched arrow* within the *coloured circle* shows the direction of rotation. The *dashed white line* oriented to the *right* stands for the resulting centrifugal force. The dashed *white line* pointing *upwards* shows the direction of the resulting flow resistance. The *solid white arrow* stands for the vectorial sum of the abovementioned forces

Cells were subjected to 24 h of fluid flow rotations at a speed level of 200 rpm. The exposed test cells within the new FSS chamber changed their orientation in accordance with the flow direction. Microscopic evaluation was conducted via a random screening at the peripheral site (2.5-cm radial distance) within a predefined area of interest (0.2 cm × 0.2 cm) through cell counting (at least *n* = 40 cells/region of interest) and morphological cell characterisation. The FSS-triggered effect was demonstrated as cells realigned themselves towards the flow direction, whereby only cells with an aspect ratio greater than 2:1 were included. To assess the alterations taking place inside the cell body, osteoblast cells were treated with a fluorescence stain to visualise actin fibres. In addition, the cells were split into two groups; the first group (*n* = 5) remained untreated, without any impact of shear stress (Fig. 4) while the cells in the second group (*n* = 5) underwent a 24-h rotational impact with 8.35 dyn/cm^2^ shear force. All tests and analysis were repeated at least six times. The cells showed a reproducible (*n* > 6) realignment of the actin cytoskeleton towards the fluid flow direction (Fig. 5), whereas the actin fibres of the untreated group showed random orientations. Findings were termed a trend if more than 50% of all screened cells (*n* > 21 cells) underwent reorientation.

## Discussion

The aim of this study was to establish a new FSS model that is easy to use as well as simple to assemble in order to create reproducible fluid shear forces on cells close to implant material surfaces. Todays’ commonly used commercial flow devices differ in geometry and function, which makes comparisons between experiments difficult [4, 10, 26, 27]. The benefits of this novel testing device are reproducible laminar flows under controlled conditions (regulated temperature as well as steady partial pressure of CO_2_). Due to its reproducibility, the stimulation of osteoblast cells by shear forces becomes assessable.

In this FSS chamber, osteoblasts were cultured on the bottom of a rotating round glass panel that moves within a resting liquid. Computerised simulations determined a value of 200 rpm as the optimal system configuration in which a constant laminar flow occurs without pulsatile character. When creating laminar flows, induction of turbulences at boundary surfaces results in flow instability. To reduce this negative effect occurring in frequently used stationary devices, cells were cultured on a carrier plate, which is placed within the lower petri dish. In this context, the direct contact between the carrier plate and another interface was omitted. Laminar flows were chosen to achieve a good reproducibility. This required a flow profile that is characterised by parallel moving liquid layers [26] that are present in the area in between the upper and lower plate. To define the most favourable position of the cell-bearing surface, computerised simulations were performed. Herein, it could be demonstrated that rising shear forces along the plate surfaces’ (0–2 dyn/cm^2^) are too low for osteoblast test cell stimulation, which occurs at about 10 dyn/cm^2^ [28, 29]. The bottom of the glass plate generated enough shear forces (10 dyn/cm^2^ in the periphery) to meet the requirements of an osteoblast-stimulating laminar flow chamber. Further on, the simulations indicated that the flow profile in between the two plates was not influenced by peripheral turbulences alongside the peripheral regions. To verify a cellular realignment towards the shear direction, cells were microscopically examined prior and after exposure to shear forces for 24 h upon a spinning disc at a speed level of 200 rpm. Even if not sufficiently meaningful alone, observing changes in osteoblast cell morphology are still appropriate methods to verify the good usability of a flow chamber for the generation of reproducible FSS. Although not statistically significant, a tendency of cellular realignment towards the liquid flow direction was demonstrated. Similarly, several studies have revealed characteristic changes of osteoblast morphology triggered by fluid shear stress, which depends on exposure time and strength [22, 30]. Likewise to our findings, these changes are characterised by the formation of actin stress fibres, which in turn align towards the longitudinal cell axis and mainly appear near the nucleus [8, 31]. However, the manual approach of analysing the actin fibres’ orientation has to be stated as a drawback of the present study, since it does not meet the requirements of a valid measurement. Immunofluorescence microscopy is largely a qualitative, or semiquantitative, approach with a limited capability of precise fibre differentiation and/or quantification since standard binary thresholds failed to exhaustively segment all fibres because of their wide variations in intensity and background levels [32]. Hence, more objective measurements could be provided by the use of an automated software-assisted processing. In this context, the FibreScore Algorithm by Lichtenstein et al. presents a potential solution for quantification, since it allows the reliable segmentation of each actin fibre. The procedure itself is based on the acquisition of different pixel intensities, whereby it works through the correlation of pixel adjacent regions with synthetic fibre templates at different orientations and their assignment for the central pixel with the highest correlation coefficient among all orientations [32].

Due to the fact that constant flows were generated within the parallel flow chamber only, the situations of in vitro experiments differ from in vivo setting where dynamic flow profiles are particular [33]. As the constant laminar flow profile is not physiological in bones [34], vessels and other tissues [35], the informative value of the experimental setting is limited but it could be used for various cell proliferation and differentiation modulations. In accordance with this, constant laminar flows were rated to have more impact on target cells than pulsatile and oscillating flow profiles. With regard to these findings, the flow profile generated within the reported device meets the requirements to induce cell morphology changes by FSS.

In addition, when using the new flow chamber, an additive effect of FSS and centrifugal forces on the cells could be seen. Other flow chambers did not reveal this phenomenon due to the fact that the liquid flow moves along a stationary cell surface only [4, 10, 22].

When comparing this new fluid flow chambers with other reported devices [4, 10], several differences are seen. Commonly, test cells are placed on a fixed surface with the culture medium flowing along. According to the method of flow generation, they can be classified into open and sealed systems. Open systems, which are hydrostatically driven, are characterised by a fluid flow that passes the stationary phase once only [36]. In sealed models, the culture medium recirculates pump driven through the system [22]. Due to their inherent system-related drawbacks such as turbulent flow generation on boundary surfaces, those flow chambers are inappropriate for laminar flow creation. However, open systems have benefits of allowing the use of different culture media in a row without ceasing the fluid shear stress. Therefore, one can easily enable or disable different stimuli by exchanging the culture medium to evaluate its cellular impact. Sealed systems such as reported in this study do not provide this option. Initially added substances within the culture medium cannot be eliminated during the experimental process. Instead, stopping the flow and draining the cells would be necessary which would cause another unwanted influence to the test cells.

Besides, in the model reported in this study, microscopic examinations are possible after completing the experiment only. Nevertheless, an advantage of the new flow chamber is the possibility of testing different cell colonies simultaneously in one single experiment by placing cells in different radial locations on the spinning disc. Due to the current flow gradient from the centre to the periphery, different cell colonies are exposed to various levels of shear forces. To simplify the process of cell reaction examination, the use of a larger sized glass panel could be considered.

Biomaterial researchers are constantly looking for innovative materials like surface-binding ligands and implant materials, pursuing the aim of improving biocompatibility and healing into host tissues. For this purpose, this new developed flow chamber could provide an easy, as well as economic way to investigate material qualities in combination with tissue cells affected by FSS. A specific material to be tested could replace the cell-bearing glass panel. Alternatively, the glass panel could be coated with surface ligands in different ways [37]. A potential use for evaluation of stem cell differentiation and/or proliferation with fluid shear stress as a mechanical stimulus may be assumed as well.

## Conclusions

To create fluid shear stress under in vitro conditions, several flow chambers have been developed in the past. The experimental setup of the flow chamber in the centre of this study offers advantages such as simplicity to assemble and ease of use as well as the creation of reproducible fluid shear forces on cells. Due to the new design, different cell types could be simultaneously analysed under reproducible conditions, by placing them in different radial positions. As a result of an increasing flow gradient from the centre to the periphery, different shear forces become available in one single experiment without changing the rotational speed level. Besides, cellular changes in osteoblast morphology and orientation using this model of fluid shear stress were proven.

## Appendix 1

**Table 1:** Listing of the single components of the flow chamber together with manufacturers’ data

Component	Manufacturer	Order no.
Large petri dish	Becton Dickinson, Franklin Lakes, NJ, USA	353025
Titanium gear shaft	Custom made	
Glass panel	Custom made	
Gear wheels	Custom made	
Electric motor	MFA Como Drills, Kent, UK	941D Series
Transformer	Voltcraft, Wollerau, Switzerland	PS 152 A

## Appendix 2

**Table 2:** Listing of the culture media and additives together with manufacturers’ data

Culture medium/additives	Manufacturer	Order no.	Concentration
Dulbecco’s modified Eagle medium (DMEM) with l-glutamine, plus 4.5 g glucose, without NaHCO_3_, without Natriumpyruvate	Life Technology, Carlsbad, USA	11966-025	
Penicillin/streptomycin	Life Technology, Carlsbad, USA	15640-055	100 IU penicillin/100 μg streptomycin/ml
Ca^2+^/Mg^2+^ free Hanks’ balanced salt solution (HBSS)–buffer	Life Technology, Carlsbad, USA	14170-070	
Fetal calf serum (FCS)	Life Technology, Carlsbad, USA	10270-098	10%
l-Glutamate	Life Technology, Carlsbad, USA	25030-024	1%
Ethylenediaminetetraacetic acid (EDTA) solution	Sigma Aldrich, St. Louis, USA	E7889	0.5 M
l-Ascorbic acid 2-phosphate	Sigma Aldrich, St. Louis, USA	A-8960	1 mmol/l ASC (affinity isolated antibody)
Dexamethasone	Serva Bioproducts, Heidelberg, Germany	18660	100 nmol/l

## Appendix 3

Phallacidin fluorescence staining

- Fixation in 4% paraformaldehyde with room temperature (10 min)
- Washing with PBS (2× 10 min)
- 0.1% Triton x-100 within PBS for cell membrane permeabilization (3–5 min)
- Washing with PBS (2× 10 min)
- Incubation with 1% PBS-BSA to avoid unspecific bindings (20–30 min)
- Incubation with Phallatoxin-solution [12.5 μl stock solution (Phallacidin B-607, Mobitec) + 500 μl PBS] (20 min)
- Washing with PBS (2× 20 min)
- Air drying of the microscope slides then covering with fluorescence mounting-medium

## Abbreviations

FSS: Fluid shear stress
